# A novel approach: enhancing marigold (*Tagetes erecta* L.) genetic transformation through seed priming technology

**DOI:** 10.3389/fpls.2024.1509720

**Published:** 2024-12-12

**Authors:** Wang-Qi Huang, Chao Meng, Lu Zhang, Feng Xu, Xiu-Mei Yang, Li-Fang Zhang, Ya-Lian Jiang, Rui-Xue Shi, A-Xiang Zhao, Yi-Ping Zhang, Niaz Ali, Xiu-Hua Chen

**Affiliations:** ^1^ National Engineering Research Center for Ornamental Horticulture, Yunnan Flower Breeding Key Laboratory, Flower Research Institute, Yunnan Academy of Agricultural Sciences, Kunming, China; ^2^ Department of Agriculture, Shaheed Benazir Bhutto University, Sheringal, Dir Upper, Pakistan; ^3^ International Agriculture Research Institute, Yunnan Academy of Agricultural Sciences, Kunming, China

**Keywords:** pigmented marigold, tagetes erecta, genetic transformation, seed priming, vacuum infiltration

## Abstract

This research presents an innovative genetic transformation protocol for marigolds (*Tagetes erecta* L.), a species of great significance in floriculture, impacting both yield and quality. The study introduces seed priming technology as a novel approach and evaluates its effect on the germination rate. The results indicate that the germination rates of pigmented marigold seeds were not significantly affected by *Agrobacterium* immersion under optimal conditions, although variations were observed in genotypes and treatment parameters. Optimal germination was observed at an optical density (OD600 nm) of 1.3 with a vacuum infiltration time of 10 min. The transgenic plants were confirmed through Basta herbicide resistance, Green florescent protein (GFP) fluorescence screening, and polymerase chain reaction (PCR) detection of the GFP gene. After the treatment, the morphological assessments showed genotype-dependent variations in plant height and fresh weight, while the biochemical analysis revealed significant variations in malondialdehyde (MDA) levels, peroxidase (POD), superoxide dismutase (SOD), and root activities. Additionally, the study examined the efficacy of various scarification techniques on seed survival rate, and seed coat removal was found to be the most effective method for marigold transformation. These findings provide a robust foundation for optimizing genetic transformation methods to enhance marigold crop resilience and quality within the floricultural sector.

## 1 Introduction

Pigmented marigold (*Tagetes erecta* L.) is a diploid species 2n=24, an annual herbaceous plant of the genus Tagetes in the family Asteraceae. It is widely grown as an ornamental plant for their vibrant colors as well as a medicinal plant, a major source of carotenoids that are extensively used for food colorants or supplements (Rodrigues et al., 2019). Among them Lutein (C_40_H_56_O_2_) is the major and abundant compound which is an essential nutrient for maintaining eye health. Its high lutein content in petals has made it an increasingly popular choice (Nagy, 2009; Sharma et al., 2024). Furthermore, lutein also possesses anti-inflammatory and antioxidant properties (Burlec et al., 2021; Nowak-Terpiłowska et al., 2023), making it an important medicinal plant. This study used *Agrobacterium* as immersion for seed priming, to infiltrate the seeds of marigold to explore the genetic transformation conditions of pigmented marigold.

The *Agrobacterium*-mediated method is the most commonly used method for plant genetic transformation (Lin et al., 2002; Sallaud et al., 2003; Chhun et al., 2007; Lin et al., 2009), which mainly relies on plant *in vitro* regeneration technology, and have about 30% transformation efficiency in rice (Lin et al., 2002). However, these methods are time consuming, laborious, requires high sterile conditions and often have problems such as, complex operation, difficulties in regeneration, somatic mutations and changed phenotypes in the subsequent generations (Franklin et al., 2004; Koetle et al., 2015). *Agrobacterium* infiltration, as an external stimulus, has a toxic effect on plant growth and development (Ziemienowicz, 2014) due to its high concentration and prolonged infusion. In the presence of external stress, plants frequently exhibit stunting and metabolite changes, such as superoxide dismutase (SOD) and peroxidase (POD) activities, however these activities decrease as the degree of stress increases (Gelvin, 2003; Burlec et al., 2021; Seleiman et al., 2021). The in planta method involves directly transforming the intact plant, without the use of callus culture or regeneration. Floral dip is a widely used in planta transformation method for *Arabidopsis* (Clough and Bent, 1998) which involves immersing flower buds directly in an *Agrobacterium* culture or dropping the inoculum of *Agrobacterium* onto the buds to facilitate contact between the bacteria and germ cells. This method is based on the responsiveness of ovule cells to *Agrobacterium* infection. In addition to *Arabidopsis*, this method is also used in several other crop species, such as wheat (Franklin et al., 2004), rice (Ratanasut et al., 2017), cowpea (Kumar et al., 2021), *Medicago truncatula* (Trieu et al., 2000), and radish (Curtis and Nam, 2001). To ensure complete contact between the transformed materials and *Agrobacterium*, external treatments such as ultrasonic treatment may be employed (Bechtold and Bouchez, 1995). Compared to the tissue-culture based *Agrobacterium*-mediated method, the in planta method is easier to use, does not require plant tissue culture, and takes less time (Kumar et al., 2021). While the in planta method has been used in various other plants, there are no reports of its use for genetic transformation via mature seed soaking (seed priming) method.

The use of *Agrobacterium tumefaciens*, a soil bacterium known for its role in plant genetic transformation, as a priming agent, is a relatively unexplored territory. The bacterium’s ability to transfer a part of its DNA to the plant cell has been harnessed in genetic engineering to introduce new traits. However, its application in seed priming could potentially offer a unique set of advantages, such as enhanced disease resistance and improved growth parameters.

There is currently no report on the effect of *Agrobacterium* soaking on the seed germination and physiological indexes of chromogenic marigolds, so relevant data from *Agrobacterium* soaking seedlings were analyzed to indirectly determine the conditions of *Agrobacterium* soaking.

The aim was to establish a simple and efficient transformation method for pigmented marigolds and optimize the best conditions for seed priming.

## 2 Materials and methods

### 2.1 Plant materials

For this study we used three genotypes of marigold; the restorer line (F), male sterile line (M), and hybrid line (F1) that were kindly provided by Yunnan Bohao Biotechnology Group Co., Ltd. Yunnan, China.

### 2.2 *Agrobacterium* culture and single colony selection

Solid YEB media supplemented with 25 mg/L of Rifampicin and 50 mg/L of Kanamycin was prepared in petri plates. The *Agrobacterium tumefaciens* strain EHA105 harboring the binary plasmid vector 3301vacGFPNM (Kunming Jin´ang Technology Co., Ltd. China) which include the neomycin phosphotransferase (*nptII*) gene (conferring resistance to Kanamycin and Neomycin) under the control of the 35S promoter, green florescent protein (GFP), and the herbicide resistant gene *Bar*, a multiple cloning site (MCS) for insertion of foreign DNA fragments, and an origin of replication (ori), responsible for plasmid replication within the host cell, was streaked onto the petri plates and incubated at 28°C overnight. Single colonies of *Agrobacterium* were selected and transferred to liquid YEB medium containing the respective antibiotics (Rifampicin and Kanamycin) and cultured to an OD600 nm= 0.5. The positive colonies were screened with PCR, where the plasmid used as positive control and ddH2O as a negative control.

### 2.3 PCR amplification and gel-electrophoresis

A total of 20 µl PCR reaction mixture was prepared as 10 µl of Master mix, 2 µl of *Agrobacterium*, 1 µl of each Forward and Reverse primer (5´TCCCACTATCCTTCGCAAGACCC3´, 5´AGTTCACCTTGATGCCGTTCTT3´, respectively) and 6 µl of water. The PCR conditions were as follow; 1 cycle of pre-denaturation at 94°C for 3 min, 25 cycles of denaturation at 94°C for 10 sec, primer annealing at 62°C for 10 sec, amplification at 72°C for 20°C, one cycle of final extension at 72°C for 2 min, and standby at 4°C. The PCR products were electrophoresed at 1% agarose gel and visualize. The positive samples were preserved by addition of 50% glyceride, frozen immediately with liquid nitrogen and stored at -80°C.

### 2.4 Resuspension of *Agrobacterium*

*Agrobacterium* EHA105 containing the vector stored at -80°C, was added to YEB medium containing 50 mg/L Kanamycin and 25 mg/L Rifampicin incubated in shaker at 28°C, 200 rpm overnight. The bacterial solution was then centrifuged at 6000 rpm for 10 min, the supernatant was discarded, and the pellet was resuspended in MS liquid medium (Solarbio, Beijing, China) consisting of MS (4.4 g/L), and sucrose (30g/L) adjusted to pH 5.9, with 0.1% Silwet L-77 and 20 mg/L Acetosyringone. The OD600 nm was measured by NanoDrop 2000 spectrophotometer (Thermo Fisher Scientific China Co., Ltd.).

### 2.5 Seed sterilization

The seeds, used in triplicate with 40 seeds per replication, were thoroughly soaked in tap water for 10-12 h, followed by sterilization with 75% ethanol by shaking for 2 min. Afterward, the seeds were rinsed twice with distilled water and subsequently sterilized with 2% sodium hypochlorite (NaClO) by shaking for 10 min. Finally, the seeds were rinsed three times with sterilized water.

### 2.6 Methods of scarification

In order to improve the germination rate, we conducted an experiment using different scarification methods, including seed coat removal, needle prick, and incised injury. The seed coat of surface sterilized seeds was carefully removed using sterile forceps. The seeds were pierced once with a sterilized needle at the tapering end, and a single small cut was made on the seeds near the tapering end. All seeds were placed on perlite filter paper in petri plates and incubated for germination.

### 2.7 *Agrobacterium* seed priming transformation

The pre-treated seeds were transferred to a beaker containing the respective resuspensions and thoroughly agitated by hand for approximately 2 min. The beakers were then placed in an ultrasonic cleaner (SK2200LHC; Shanghai Kedo Ultrasonic Instrument Co., Ltd.) operating at 53 kHz for 15 min to remove any bubbles and ensure full contact between the seeds and the immersion solution. Subsequently, the samples were kept at room temperature for 2 min before being placed in a vacuum chamber (Nalgene 5310-0250) for 5, 10, and 15 min, respectively. Following each treatment, a 30 sec vacuum treatment was performed to minimize negative atmospheric pressure. The seeds were then transferred to a shaker at 25°C and 150 rpm for 4 h. After shaking, the treated seeds were transferred to filter paper to absorb the surface liquid (excess bacterial immersion) and then placed on wet filter paper in petri plates. The plates were incubated at 25°C for 48 h under controlled environmental conditions with a 16/8-hour light/dark photoperiod, 60-70% relative humidity, and 2000-3000 lux light intensity to allow for germination. The seed germination rate was observed and recorded according to the following formula.


$$Germination\;rate\;\left(GR\right)=\frac{Seeds\;germinated\;\;}{Total\;seeds\;sown}\times 100$$

### 2.8 Screening of transgenic plants using GFP vector

To screen transgenic plants using the GFP vector, 15-days-old seedlings were utilized. Various parts of the seedlings, including cotyledons, hypocotyls, and roots, were observed under a laser scanning microscope (Leica MZ16 fluorescence stereomicroscope) equipped with a GFP filter (LUYOR-3415 Shanghai Luyang Biotechnology Co., Ltd.). A Dual Fluorescent Protein Flashlight served as the excitation light source and photographed accordingly. Positive plant samples were subsequently excised and placed into centrifuge tubes, followed by immersion in liquid nitrogen for 5 min to facilitate lyophilization. The samples were then ground using a grinding rod to prepare them for RNA extraction.

### 2.9 RNA extraction and RT-PCR

RNA was extracted from the positive plants detected by green fluorescence using the TRI-zol method according to the manufacturer’s protocol (Rio et al., 2010; Singhal and Bose, 2024). First-strand cDNA synthesis was performed using 2 µg of RNA with the PrimeScript II 1^st^ strand cDNA Synthesis Kit (6210A, Takara, Dalian, China) following the manufacturer’s instructions and as previously described (Chen et al., 2016). The cDNA was used for the identification of transgenic positive plants.

### 2.10 Phenotypic characterization

The 23-25-days-old seedlings were transplanted into 50-cells plug trays filled with moistened soil (Lusen, nutrient soil, Jiangxi, China). During “two leaves and one heart” stage, which typically occurs in 23- to 25-day-old plants, the seedlings were transferred to nutrient-rich soil in the plug trays. At this stage, various seedling indicators, including plant height, fresh weight, and root length, were measured.

### 2.11 Biochemical characterization

Biochemical indexes were measured in plants at the “two leaves and one heart” stage of seedlings. Superoxide dismutase (SOD) activity was determined using the nitrogen blue tetrazolium method (Sun et al., 1988; Senthilkumar et al., 2021a), malondialdehyde (MDA) content was determined using the thiobarbituric acid colorimetric method (Senthilkumar et al., 2021b; Malondialdehyde (MDA) colorimetric assay kit, 2024), and peroxidase (POD) activity was determined using the guaiac lignanol method (Kumar et al., 2021). The root viability was determined using the TTC (triphenyl tetrazolium chloride) method (Trieu et al., 2000).

### 2.12 Exploration of injury/scarification techniques

Seeds that underwent pretreatment were examined for various scarification approaches, including seed coat removal, needle stick injuries, and cutting injuries. The germination rate was calculated by placing the seeds on moistened filter paper.

### 2.13 Basta Screening determination

After seed priming with agroinfiltration, the seed coat was removed, and 40 seeds for each transgene (*Agrobacterium* infected) and control (without *Agrobacterium* infection) in three replications, were placed on perlite filter paper containing a high concentration (200 mg/L) of Basta. The survival rate was recorded after germination to screen out Basta-resistant positive plants.

### 2.14 Statistical analysis

For data analysis and ANOVA, SPSS 21.0 software was used. OriginPro 2021 was employed for plotting, while Microsoft Excel 2019 was used for statistical analysis, including standard deviation calculation and bar graph creation.

## 3 Results

### 3.1 Effect of *Agrobacterium* infiltration on germination rate of pigmented marigold

**Figure 1:**
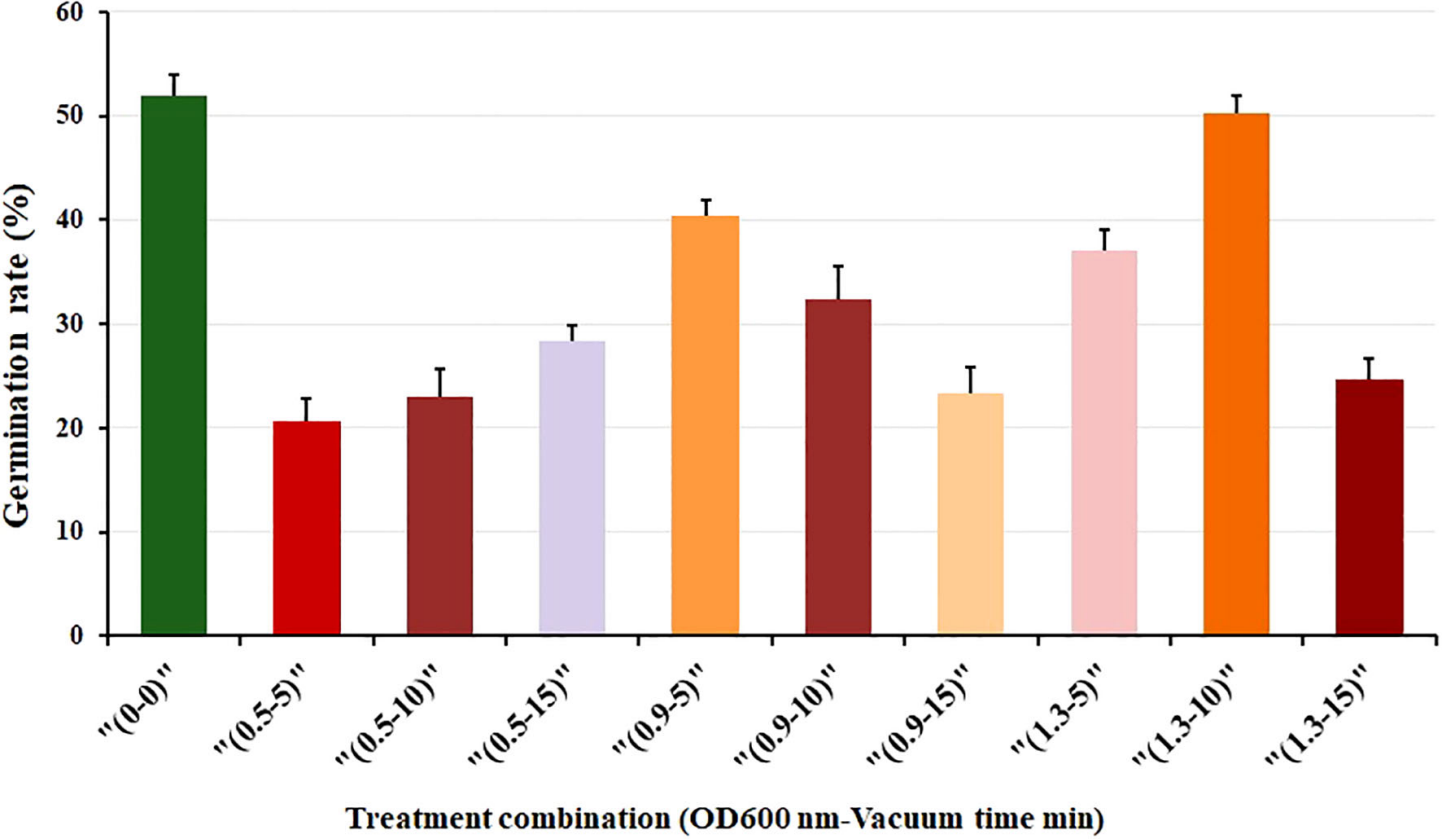
Seed germination rate (%) of F1 genotype under various combinations of immersion concentration (OD600 nm) and vacuum time (min). Each treatment was conducted with 40 seeds, in three replications. Error bars represent the standard deviation (SD).

We investigated seed germination by subjecting seeds to various combinations of *Agrobacterium*-mediated immersion concentrations (measured as OD600 nm) and vacuum exposure times (in minutes). Ten treatment combinations, including a control group, were evaluated in triplicate. Three genotypes; F1, F, and M, were screened and tested for germination rate. Among these, genotype F1 consistently exhibited favorable germination results and was subsequently selected for further evaluation of the combined effects. In the control treatment (0–0), where no bacterial immersion or vacuuming occurred, the average germination rate was 52% (**Figure 1**). Different treatment conditions yielded varying results. The highest germination rate (50%) was observed under the condition of 1.3 nm and 10 min (1.3-10). Additionally, the treatment with 0.9 nm and 10 min (0.9-10) also produced a similarly high germination rate (40%). Conversely, the lowest germination rate occurred at 0.5 nm and 5 min (0.5-5), followed closely by 1.3 nm and 15 min (1.3-15) as shown in **Figure 1**. Furthermore, our observations revealed an initial enhancement in germination rate with increasing immersion concentration and vacuum exposure time. Specifically, at a concentration of 0.5 nm, a longer vacuum time led to improved germination (**Figure 1**). In contrast, a combination of 0.9 nm after 5 min (0.9-5) resulted in a higher germination rate. However, germination rates declined as vacuum exposure time increased. Conversely, 1.3 nm exhibited varied germination rates: an initial increase up to 10 min of vacuum treatment, followed by a sharp decline at 15 min (**Figure 1**).

These findings indicate the significant impact of the immersion concentration and vacuum exposure time combination on seed germination, emphasizing the need for precision in experimental design. These findings suggest that precise *Agrobacterium* concentration and optimal vacuum timing do not negatively impact seed germination. This method can be effectively employed for genetic transformation while maintaining good seed germination rates.

### 3.2 Identification of transgenic plants with basta applications

**Figure 2:**
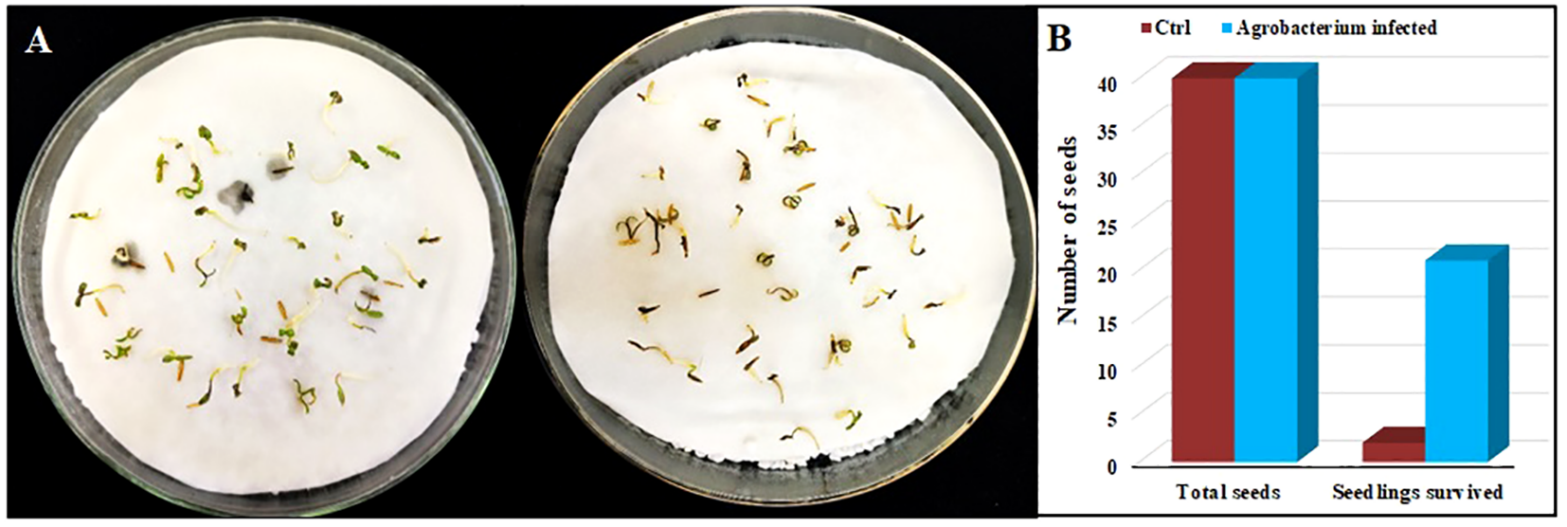
Screening of transgenic plants through herbicide basta (200 mg/L) application. **(A)** Transgenic (*Agrobacterium*-infected) seeds (left) and control (without *Agrobacterium* infection) seeds (right) were photographed 10 days after basta application. **(B)** Number of total seeds and the survival rate after basta application.

Upon exposure to varying concentrations of Basta, seeds containing the binary vector with the screening gene for Basta herbicide resistance were tested. As the concentration of Basta increased, the survival rate of the seedlings declined, with a survival rate of 1.6% observed when the concentration reached 200 mg/L in control seedlings. Subsequently, high concentrations of Basta (200 mg/L) were applied to both *Agrobacterium*-infected and control (without *Agrobacterium* infection) plants. Generally, marigold plants require approximately 23 to 25 days from the time of bacterial infection to the transfer of seedlings to soil for growth. The results showed that while the non-transgenic plants almost died, the transgenic seedlings survived after being treated with 200 mg/L of Basta (as shown in **Figure 2**).

### 3.3 Screening of positive plants using gfp florescence

**Figure 3:**
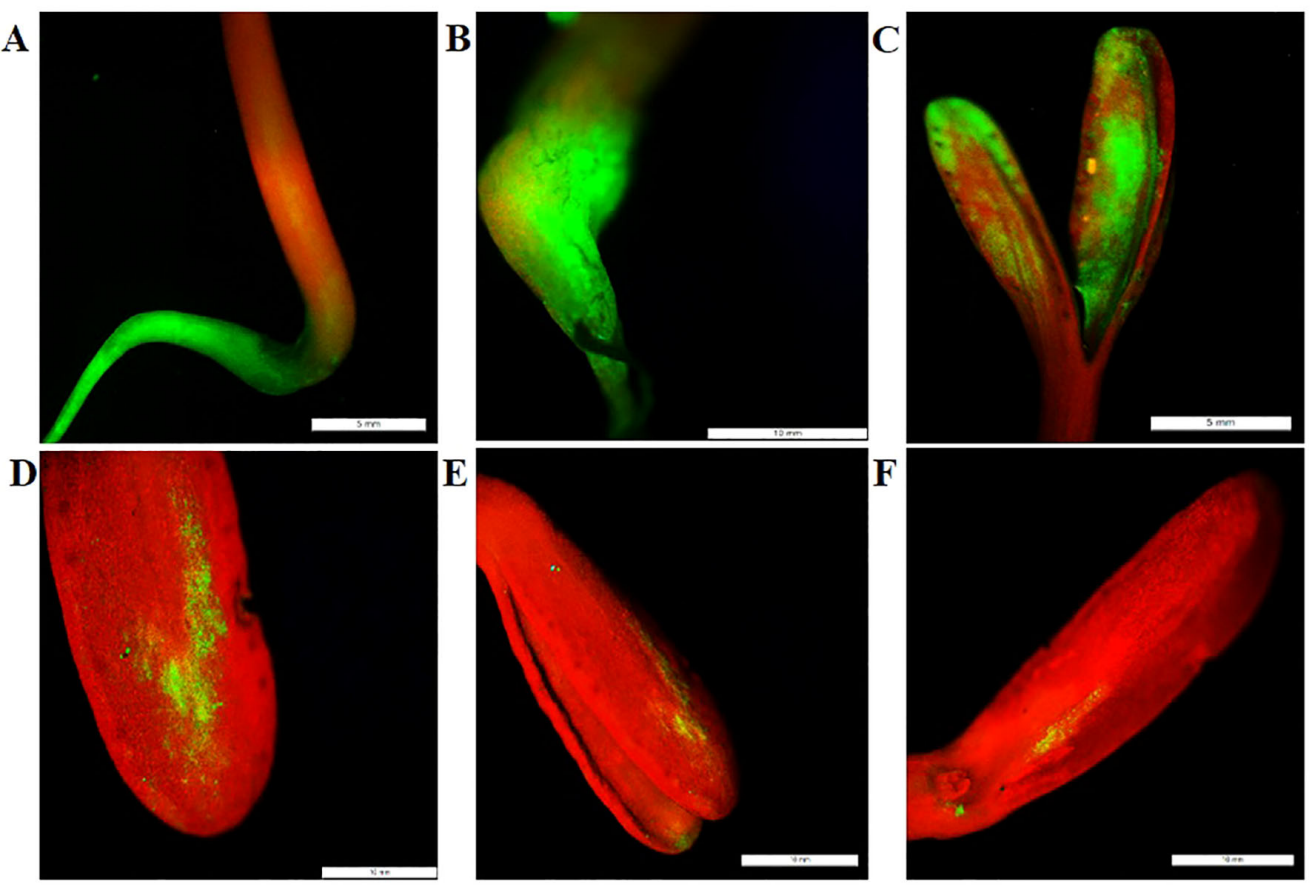
GFP Expression Analysis in Transgenic Marigold Seedlings. Detection of GFP expression was performed using a Fluorescence Stereo Microscope equipped with a GFP filter. GFP fluorescence was observed in various parts of the seedlings: **(A)** root, **(B)** hypocotyl, and **(C–F)** cotyledons from different plants. Scale bar represents 5 mm in **(A, C)**, and 10 mm in **(B, D–F)**.

In order to further identify the positive plants, a GFP filter was utilized. The Green fluorescence was detected in various parts of the seedlings, including the root, hypocotyl, and cotyledons, as shown in **Figure 3**.

### 3.4 Confirmation of transgenic plants with reverse transcription-polymerase chain reaction

**Figure 4:**
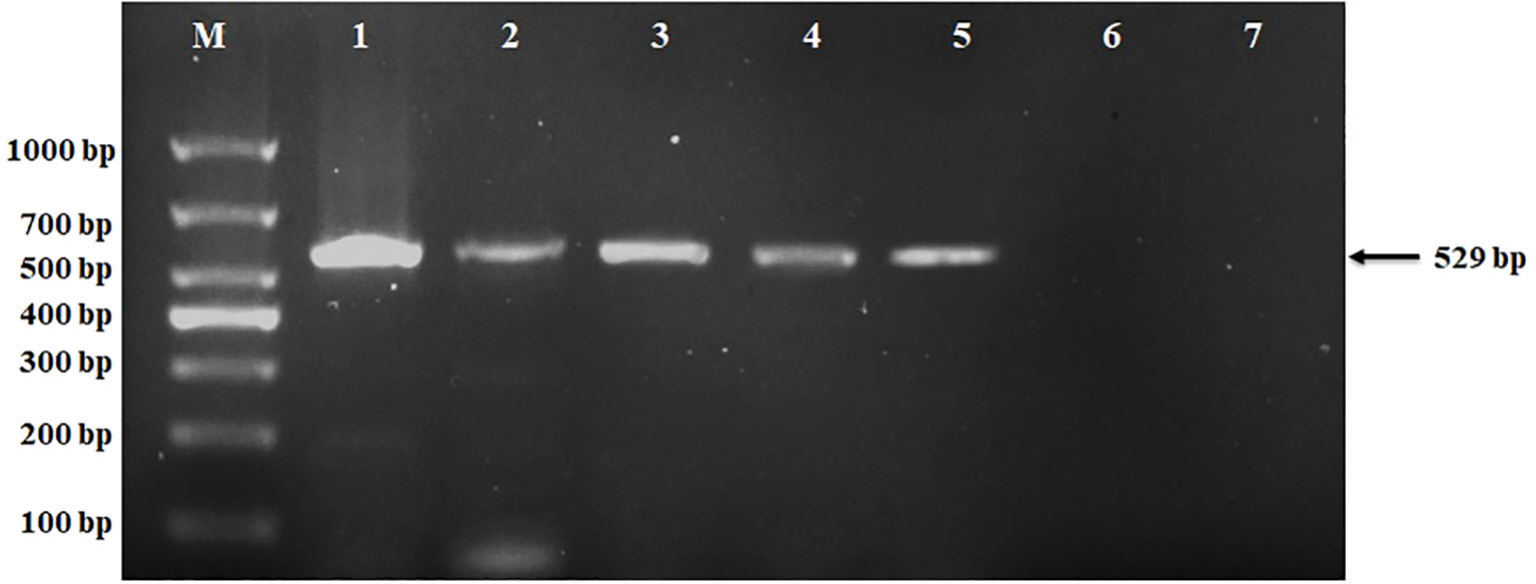
Detection of positive plants with PCR. Lane M: 1000 bp marker;1: Plasmid/positive control; 2-5: transgenic plants 6: Ctrl non-transgenic control plants; 7: Ctrl PCR blank as negative control.

The binary vector 301vacGFPNM, containing the reporter gene GFP, selectable marker gene Basta, and 3X 35S promoter, was successfully transformed into marigold seeds using seed priming technology. To confirm the genetic transformation in marigold (*Tagetes erecta* L.), RNA was extracted from both transgenic and non-transgenic control plants and reverse transcribed into cDNA. PCR was performed using gene-specific primers for the GFP reporter gene, resulting in the amplification of a 529 bp fragment in the transgenic plants. A plasmid containing the GFP gene served as a positive control, while non-transgenic control plants and a PCR blank (without DNA) were used as negative controls, as depicted in **Figure 4**. The method was applied to three genotypes: F, F1, and M. All genotypes produced transgenic plants; however, based on germination frequency and consistency results, we focused on the F1 genotype. **Figure 4** illustrates the transgenic plants of the F1 genotype, validating our transformation method.

### 3.5 Effect of *Agrobacterium* seed immersion on morphological indexes of pigmented marigold seedlings

**Figure 5:**
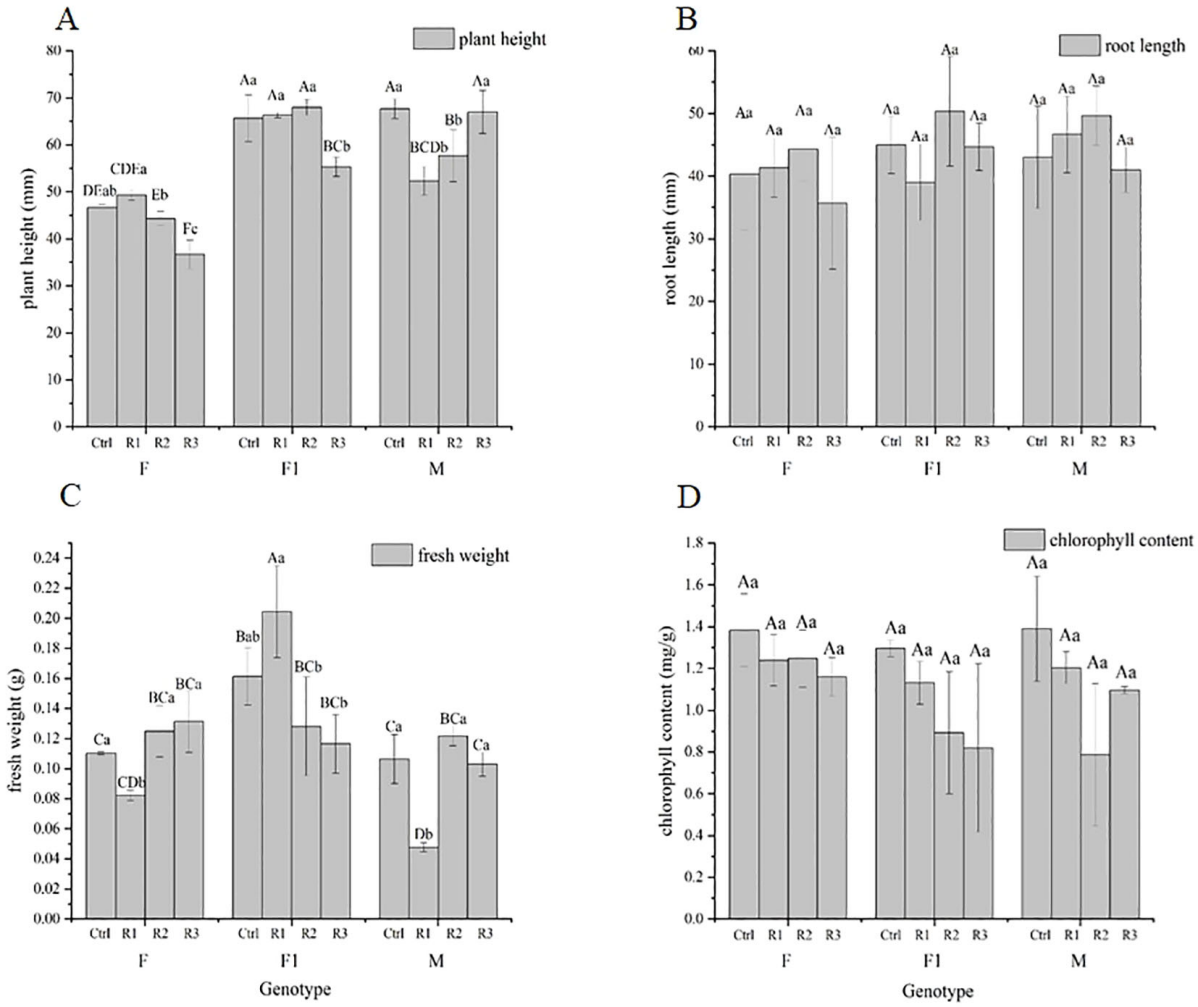
Effect of *Agrobacterium* seed immersion on morphological indexes of pigmented marigold. **(A)** Plant height **(B)** root length **(C)** fresh weight and **(D)** chlorophyll content. The uppercase letters in the figure show the differences among treatments, and the lowercase is the differences within the same species. Control (Ctrl) represents without *Agrobacterium* infection and non-vacuumed plants (0-0). Treatment groups include R1 (0.5-5), R2 (0.9-10), and R3 (1.3-15).

Differences in plant height and fresh weight were observed among various genotypes after treatment, as well as within the same genotype after different treatments (**Figure 5**). Significant differences were noted within the same genotype under varying treatment conditions. In terms of plant height, the effect of *Agrobacterium* immersion on each genotype was F ≈ F1 > M. Overall, the effects of various conditions on plant height were genotype > vacuum treatment time > infection concentration, with the influence of infection *Agrobacterium* concentration and vacuum treatment time being more pronounced on F (**Figure 5**). For fresh weight, the effect of *Agrobacterium* immersion on each genotype was F1 > M > F, and the effects of various conditions on fresh weight were genotype > vacuum treatment time > infection concentration. There were no significant differences in root length or chlorophyll content among genotypes, and no significant differences were observed in root length or chlorophyl content under each treatment of the same genotype. These results suggest that genotypes, vacuum treatment time, and infection *Agrobacterium* concentration have interactive effects on plant height and fresh weight but have little effect on root length and chlorophyll content.

### 3.6 Effect of *Agrobacterium* seed immersion on biochemical indexes of pigmented marigold

We observed that the malondialdehyde (MDA) content in genotypes F and F1, after being soaked in *Agrobacterium*, was significantly higher than that in genotype M. The impact of *Agrobacterium* immersion on each genotype followed the order: F > F1 > M. Although there was no significant difference in the MDA content of marigold after *Agrobacterium* immersion, the effect was most pronounced in genotype F. The influence of the three conditions on MDA content was ranked as follows: genotype > immersion concentration ≈ vacuum treatment time.

In terms of peroxidase (POD) activity, genotype F exhibited significantly higher POD activity compared to genotypes M and F1 after *Agrobacterium* soaking. The impact on each variety was ranked as follows: F > F1 > M. The effects of the three conditions on POD activity were as follows: genotype > vacuum treatment time ≈ infection concentration.

Regarding superoxide dismutase (SOD) activity, there was a significant difference in SOD activity between the treatments after *Agrobacterium* immersion. The effect on each genotype was ranked as follows: F > F1 > M. *Agrobacterium* seed soaking had a greater impact on genotype F and a lesser impact on other genotypes. The influence of the three conditions on SOD activity was ranked as follows: genotype > vacuum time > immersion concentration.

There was a significant difference in root activity between various genotypes after *Agrobacterium* immersion, and a significant difference was also observed between different treatments of the same genotype. The effect on each genotype was ranked as follows: M > F1 > F. *Agrobacterium tumefaciens* immersion had a substantial influence on the root activity of each genotype. The impact of the three conditions on root activity was ranked as follows: infection concentration > genotype > vacuum.

**Figure 6:**
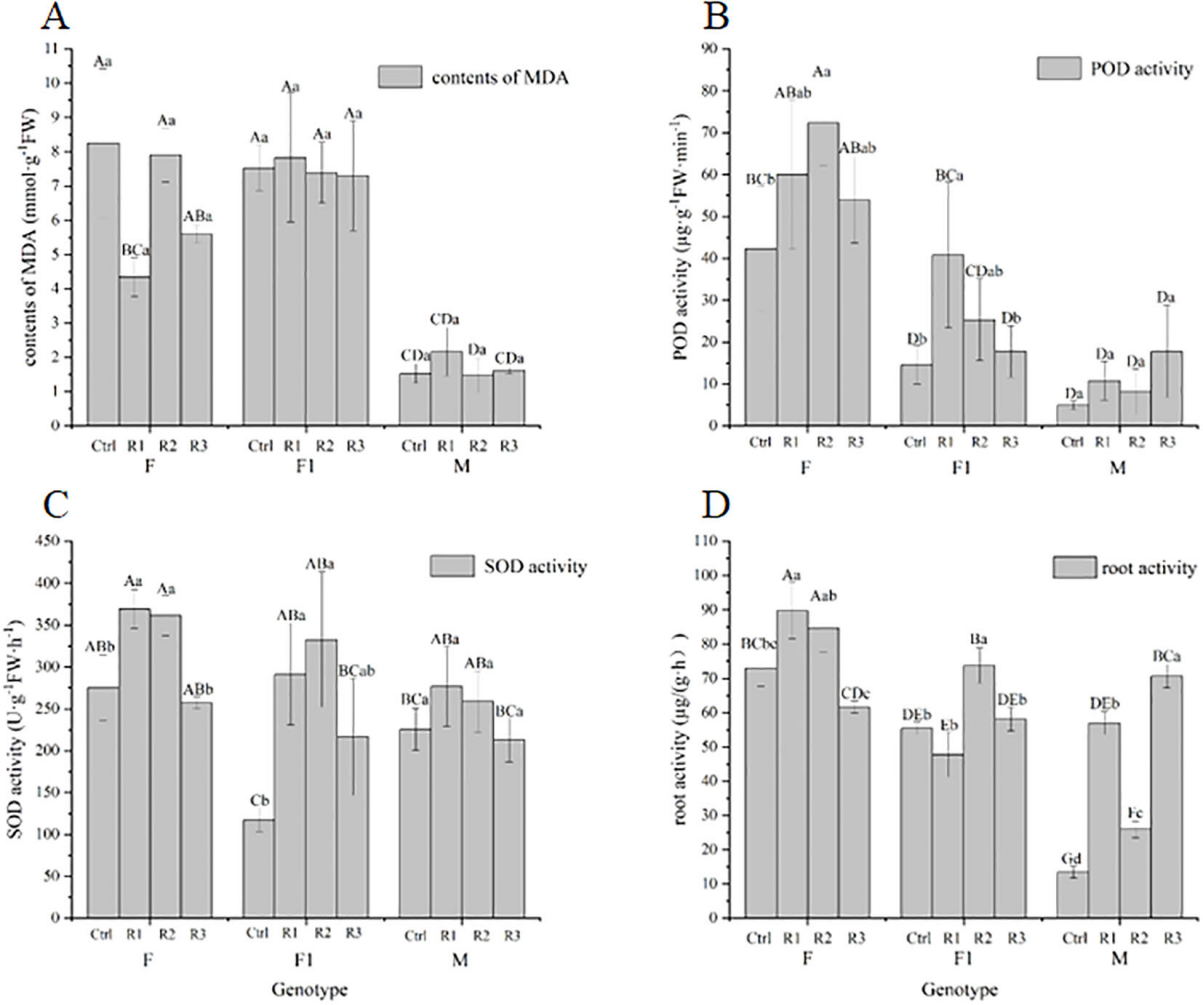
Effect of *Agrobacterium* seed immersion on biochemical indexes of pigmented marigold **(A)** contents of MDA **(B)** POD activity **(C)** SOD activity **(D)** root activity. Same as in **Figure 5**.

From **Figure 6**, it can be observed that significant differences in MDA content, POD activity, SOD activity, and root activity were present among different genotypes after treatment, as well as within the same genotype after different treatments. These results suggest that genotype, vacuum treatment time, and immersion concentration have interactive effects on MDA content, POD, and SOD activity.

### 3.7 Effect of different scarification methods on seed survival rate

**Figure 7:**
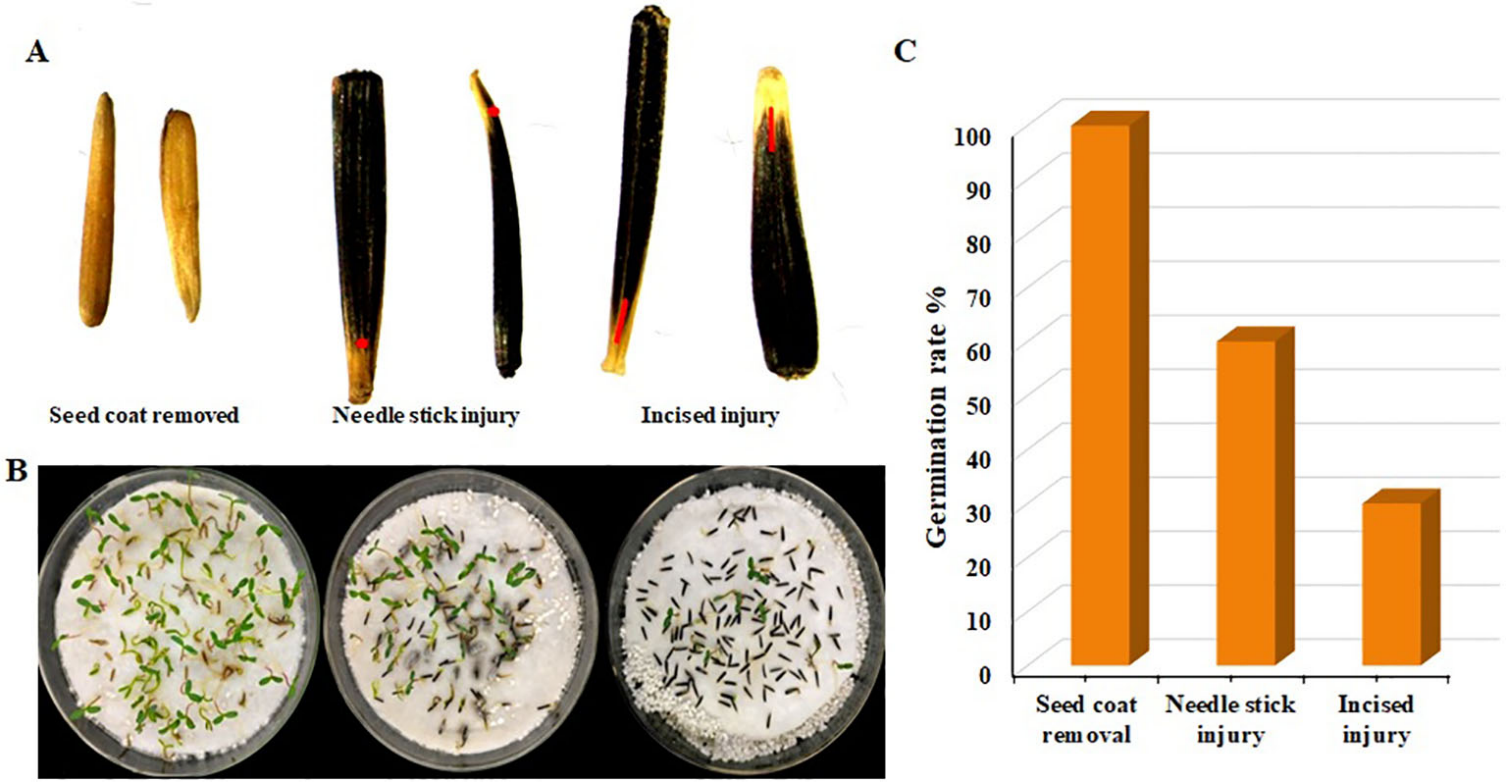
Seed germination with three different scarification methods. **(A)** Seeds with scarification method used **(B)** Seed germination **(C)** Germination rate. Red point indicates scarified area.

*Agrobacterium* possesses the attributes of infected wounds, and a certain level of damage to the impregnated material can enhance the transformation efficiency. However, due to the diverse ways of wounding/scarification, the damage to the seeds and the germination rate varies. Through the analysis of the germination rate of F seeds, a suitable method for creating wounds in marigold seed transformation was identified. The findings demonstrated that the seed germination rate was highest in the removed seed coat, with almost all seeds germinating, followed by a germination rate of approximately 60% for needlestick wounds, and the lowest germination rate of around 30% for cut wounds (**Figure 7**). In conclusion, the removal of seed coats is a suitable method for creating wounds in marigold soaking.

## 4 Discussion

Yield and quality are the main parameters for the high demands of floriculture. The advent of climate change exerts profound effects on horticultural crops, encompassing both ornamental and medicinal varieties. These impacts manifest through alterations in phenological stages, increased disease incidence, and changes in the biochemical composition of crops (Bisbis et al., 2019; Mandal and Lalduhsangi, 2020; Akhtar et al., 2021; Pawar and Sakhale, 2024). To counteract these climatic challenges, the development of stress-resilient cultivars is imperative. In the context of marigold breeding, traditional crossbreeding techniques are often protracted and constrained in generating the desired genetic variation. Genetic engineering emerges as an efficient alternative, offering a streamlined route to enhance marigold traits. Various studies have explored genetic transformation techniques, including tissue-culture based approaches using *Agrobacterium* for *in vitro* regeneration and transformation (Zhang et al., 2000; Nuoendagula et al., 2017), particle bombardment (Vanegas et al., 2006) and floral dip methods (Cheng et al., 2019). A novel in planta particle bombardment (iPB) method, pioneered by Hamada et al., (Hamada et al., 2017), involves the extraction of embryos from mature seeds for bombardment via a biolistic gun, followed by cultivation on growth media. Despite its innovation, this technique is marred by complexity, suboptimal efficiency, elevated costs, and extensive time requirements. Previous genetic transformation strategies are similarly beleaguered by issues such as regeneration difficulties, high expenses, and lengthy processes.

Vacuum infiltration technique, initially applied to *Arabidopsis (* Bechtold and Bouchez, 1995), yielded a mere 1% success rate in generating transgenic plants, this method has been employed in several other species, including *Petunia hybrid (* Tjokrokusumo et al., 2000), *Medicago truncatula (* Trieu et al., 2000), *Brassica rapa* L. ssp. *Chinensis* (Liu et al., 1998; Qing et al., 2000), *Arabidopsis laciocarpa* (Tague, 2001), and *Raphanus sativus* L. *longipinnatus Bailey* (Curtis and Nam, 2001; Curtis et al., 2002). However, its applicability is hindered by its dependency on plant morphology and size, rendering it unsuitable for larger plant species. Transformation techniques involving flowers (Bechtold and Bouchez, 1995; Clough and Bent, 1998) and seedlings (Trieu et al., 2000) have been successfully implemented across a diverse range of plant species. In contrast, seed transformation represents an innovative approach that has yet to be characterized.

In our investigation, we utilized the mature seeds of marigold (*Tagetes erecta* L.), thereby circumventing the need for whole-plant usage or *ex vivo* embryo treatment, as shown in **Figure 7**. By amalgamating seed priming techniques with vacuum infiltration, we ensured comprehensive contact between the *Agrobacterium* inoculum and the seed’s cellular structure. The transgenes were confirmed through PCR amplification in the T0 generation and further validated in T1 plants (**Figure 4**), demonstrating stable transformation in marigolds. Our methodology proved to be efficient, straightforward, cost-effective, and devoid of tissue culture requirements, showing promise for application in other species, including those recalcitrant to tissue culture.

The acceleration of protein-nucleic acid hydrolysis results in a decrease in chlorophyll content, oxidation of the protoplasmic and inner membrane systems, an increase in the content of oxidation product MDA, and a decrease in the activity of the POD defense system (Afzal et al., 2005; Singh et al., 2007). This study has demonstrated that the MDA content of pigmented marigolds increased at low concentrations for a short period, but then began to decrease after reaching a certain threshold, differed from the findings of Wang et al (Wang et al., 2023). This variation may be attributed to the fact that POD activity initially increases and then decreases with an increase in immersion concentration, which is consistent with the results of Tian et al., (Tian et al., 2012). In certain varieties, SOD activity initially increases and then decreases with an increase in immersion concentration and vacuum time (**Figure 6**), which is consistent with the results of Tian et al., (Tian et al., 2012), while the genotype M is not sensitive to changes in SOD activity after treatment.

Optimum immersion concentration and vacuum time are crucial for establishing an *Agrobacterium*-mediated seed priming transformation system, especially for sensitive varieties. In *Agrobacterium*-mediated seed priming genetic transformation, excessively high immersion concentration or prolonged vacuum time may cause irreversible damage to seeds, while very low concentrations or short times may result in insufficient contact between *Agrobacterium* and seeds, thus affecting transformation efficiency. Trieu et al., (Trieu et al., 2000), used this method in Medicago seedling and got 9.2% transformation efficiency. Lin et al., (Lin et al., 2009) used a bacterial immersion of OD600 nm= 0.6 and 15 min of vacuum time resulted in 6% of transformation frequency. Zhao (2024) et al., discovered that an OD600 nm immersion concentration of 0.35 provided the best results for transforming white clover. Lin et al., (Lin et al., 2009), discovered that the needle piercing method of scarification in indica rice seeds could enhance seed transformation efficiency. These findings demonstrate that there are differences in immersion concentrations and vacuum times among various materials and methods during the transformation process (**Figure 1**), and different scarification techniques also impact seed transformation efficiency (**Figure 7**).

The findings of this study revealed significant variations in the sensitivity of different genotypes to immersion concentration and vacuum time. Specifically genotype F was more sensitive to *Agrobacterium* immersion than F1 and M. Genotype F demonstrated the stress phenomenon of seedlings at low immersion concentration and short vacuum time. High immersion concentration (>1.3) or long vacuum time (>15 minutes) treatment caused irreversible damage to them. After a comprehensive analysis of the determination standard, F1 was chosen as the material in the bacterial solution with an OD600 nm of 1.3, and vacuum time of 10 min was deemed optimal for transformation in pigment marigold seeds (**Figure 1**).

*Agrobacterium*, a commonly used tool in plant transformation, contains a specific transfer sequences (T-DNA) region. When *Agrobacterium* infects an injured part of a plant, genes in the virulence (vir) region can be induced by phenolic and carbohydrate substances secreted by the plant. The proteins encoded by these genes cleave the T-DNA region from the tumor-inducible plasmid or the root-inducible plasmid and deliver it into the plant cell as single-stranded DNA. Therefore, proper wound fabrication of impregnated materials can improve the transformation efficiency. However, an unsuitable wound method can easily lead to the loss of seed vitality. The author has explored the best scarification method for the transformation of *Agrobacterium* marigold by removing the seed coat as shown in **Figure 7**.

## Conclusion

The findings outline the potential of genetic engineering as a viable alternative to traditional breeding methods for enhancing marigold traits. It discusses various genetic transformation techniques, with a focus on a novel seed transformation approach that bypasses the need for whole-plant usage or *ex vivo* embryo treatment. This method combines seed priming with vacuum infiltration, proving to be efficient, cost-effective, and suitable for species resistant to tissue culture. The study also emphasizes the importance of optimizing immersion concentration and vacuum time to achieve successful transformation without damaging the seeds. The findings suggest that this innovative technique could revolutionize the genetic transformation of marigolds and potentially other plant species.

## Data Availability

The original contributions presented in the study are included in the article/supplementary material, further inquiries can be directed to the corresponding author/s.
